# Olfactory Network Functional Connectivity as a Marker for Parkinson’s Disease Severity

**DOI:** 10.3390/life15081324

**Published:** 2025-08-20

**Authors:** Senal Peiris, Anupa Ekanayake, Jiaming Lu, Rommy Elyan, Katie Geesey, Ross Cottrill, Paul Eslinger, Xuemei Huang, Prasanna Karunanayaka

**Affiliations:** 1 Department of Radiology, The Pennsylvania State University College of Medicine, Hershey, PA 17033, USA; speiris@pennstatehealth.psu.edu (S.P.); aekanayakemudi@pennstatehealth.psu.edu (A.E.); b101230039@gmail.com (J.L.); relyan@pennstatehealth.psu.edu (R.E.); kgeesey@pennstatehealth.psu.edu (K.G.); rcottrill@pennstatehealth.psu.edu (R.C.); peslinger@pennstatehealth.psu.edu (P.E.); 2 Medical School of Nanjing University, Nanjing University, Nanjing 210993, China; 3 Department of Neurology, The Pennsylvania State University College of Medicine, Hershey, PA 17033, USA; 4 Department of Neurology, School of Medicine, The University of Virginia, Charlottesville, VA 22903, USA; xhuang3@pennstatehealth.psu.edu

**Keywords:** UPSIT, resting-state fMRI, olfactory network, functional connectivity, Parkinson’s disease

## Abstract

Olfactory impairment was assessed in akinetic-rigid (PD_AR_) and tremor-predominant (PD_T_) subtypes of Parkinson’s disease (PD), classified based on motor symptoms. Seventeen PD_AR_, fifteen PD_T_, and twenty-four cognitively normal (CN) participants completed the University of Pennsylvania Smell Identification Test (UPSIT). Groups were well-matched for age and demographic variables, with cognitive performance statistically controlled. Resting-state fMRI (rs-fMRI) and seed-based functional connectivity (FC) analyses were conducted to characterize olfactory network (ON) connectivity across groups. UPSIT scores were significantly lower in PD_AR_ compared to PD_T_. Consistently, ON FC values were reduced in PD_AR_ relative to both PD_T_ and CN. FC of the primary olfactory cortex (POC) significantly differed between CN and the PD subtypes. Furthermore, connectivity in the orbitofrontal cortex and insula showed significant differences between PD_AR_ and PD_T_, as well as between PD_AR_ and CN. Notably, ON FC between the left hippocampus and the posterior cingulate cortex (PCC) also differed significantly between PD_AR_ and PD_T_. These findings reveal distinct ON FC patterns across PD_AR_ and PD_T_ subtypes. Variations in UPSIT scores suggest that motor symptom subtype is associated with olfactory performance. Moreover, ON connectivity closely paralleled the UPSIT scores, reinforcing a neural basis for olfactory deficits in PD. Given the accelerated motor and cognitive decline often observed in the PD_AR_, these results support the potential of olfactory impairment as a clinical marker for disease severity.

## 1. Introduction

Parkinson’s disease (PD) is marked by progressive motor symptoms, including tremors, rigidity, and bradykinesia [1,2]. Variability in symptom presentation has led to classification into subtypes, notably akinetic-rigid (PD_AR_) and tremor-predominant (PD_T_) [3,4,5]. Among these, PD_AR_ is associated with poorer clinical outcomes and more rapid cognitive decline [6,7,8,9]. Importantly, motor symptoms are often preceded by a range of non-motor symptoms, sometimes emerging years earlier [10,11]. Impaired olfaction is one such early symptom and is considered a predictive marker of cognitive decline and neurodegeneration in PD [12,13,14,15]. Specifically, this impairment has been partly attributed to olfactory bulb (OB) degeneration, alpha-synuclein deposition, and the loss of mitral and tufted cells [12].

Motor symptoms in PD are primarily linked to dopaminergic cell loss in the nigrostriatal pathway [16], whereas olfactory impairment—a common preclinical symptom in de novo PD—is associated with extranigral pathology [17,18]. Despite both being predictive of PD dementia [10,19], the relationship between motor and olfactory symptoms remains unclear. However, behavioral studies suggest more pronounced olfactory deficits in PD_AR_ compared to PD_T_ [20,21]. For instance, Solla. et al. (2021) reported greater odor threshold impairment in PD_AR_ [20].

Previously, Karunanayaka et al. (2016) used resting-state fMRI (rs-fMRI) from this cohort to examine default mode network (DMN) differences in PD [22]. Another rs-fMRI study explored neuro-temporal functional connectivity (FC) patterns associated with olfactory dysfunction in PD, Alzheimer’s disease (AD), and mild cognitive impairment (MCI) [23]. In healthy adults and older individuals, the sense of smell has been shown to correlate with cognitive performance [24,25]. In contrast, the current study focuses specifically on olfactory network (ON) FC differences between PD_AR_ and PD_T_ subtypes—a distinct aim not addressed in our prior work. Any potential overlap with previous studies is acknowledged and clarified in the Section 4.

This study investigated whether the neural substrate underlying olfactory function is differentially affected in the PD_AR_ and PD_T_ subtypes [26], which were well-matched for age, demographic variables, and cognitive performance. Olfactory performance was measured using the UPSIT [27], while rs-fMRI data were used to examine ON FC. We hypothesized that ON FC would differ between PD_AR_ and PD_T_, and that these differences would correspond with variations in UPSIT performance.

## 2. Methods

### 2.1. Study Subjects

In total, 17 PD_AR_, 15 PD_T_, and 24 CN subjects were recruited for this study. Based on the G*Power 3.1.9.7 analysis conducted for a one-way analysis of variance (ANOVA) with fixed effects, the required sample size was calculated to achieve a power of 0.95 (β = 0.05) with an effect size of f = 0.6, a significance level of α = 0.05, and 3 groups. The analysis determined that a total sample size of 48 (16 per group) would yield an actual power of approximately 0.958. However, our study’s sample size of 56 participants exceeds this requirement, ensuring even greater statistical power to detect the hypothesized effect. The results confirm that the sample size is sufficient to reliably test the hypothesized group differences under the specified parameters, reducing the risk of a Type II error and enhancing the robustness of our findings. The samples were matched as closely as possible for age, sex, education, and cognitive status (Table 1). PD_AR_ and PD_T_ subjects were additionally matched for disease severity via standard clinical scales. Both the PD cohort and the CN subjects, including spouses and relatives of PD participants, were recruited from a tertiary care movement disorder clinic and were part of NIH-funded PD biomarker studies at the Penn State Hershey Medical Center. As previously stated, a description of the data used in this study can also be found in Karunanayaka et al. (2016) [22].

The protocols of this study strictly followed the principles of the Declaration of Helsinki [28] and were also approved by the Institutional Review Board (IRB) of the Penn State Hershey Medical Center. As stipulated in the IRB approval, informed, written consent was obtained from each subject before taking part in this study between the year 2009 and 2014.

All PD subjects were assessed by a movement disorder specialist following the criteria outlined in Calne et al. (1992) [29]. Two PD subjects had very mild symptoms and were drug-naïve; all others were treated with antiparkinsonian medications. PD_AR_ and PD_T_ subjects had no other health issues including neurological disorders, hypothyroidism, otolaryngological diseases, vitamin B12 and folate deficiencies, and kidney or liver diseases. Only right-handed PD subjects less than 70 years of age with a Mini-Mental State Examination (MMSE) score of at least 24, and who took neither a centrally acting acetylcholinesterase inhibitor nor memantine, were included in this study. Subjects completed neuropsychological testing (summary provided in Supplementary Table S2) to confirm a normal range of cognition as well as screening for depression. CN subjects had no history of neurological or psychiatric disorders, or previous head injuries. CN and PD subjects were pre-screened for possible MRI compatibility complications such as metal implants and claustrophobia.

### 2.2. PD_AR_ and PD_T_ Classification

PD subjects were classified as either PD_AR_ or PD_T_ using the modified ratio developed by Schiess et al., which is based on the UPDRS III, with a numerical ratio derived from mean tremor and akinetic-rigidity scores [8,30]. The tremor was assessed using a nine-item scale, which included a history of left or right arm tremors (two items); rest tremors of the face, lips, chin, and each limb (five items); as well as postural tremors of the right and left upper extremities (two items). The 14-item akinetic-rigidity scale assessed the passive range of motion: the rigidity of the neck and at each extremity (five items), rapid opening and closing of the hands (two items), finger tapping (two items), rising from a chair (one item), posture and postural instability (two items), gait (one item), and body bradykinesia (one item). Each item was rated from 0 to 4, with zero representing an absence of symptoms (or normal activity), and 4 indicating the presence of significant symptoms or impairments. The mean of each scale was calculated and then the ratio (tremor/akinetic rigidity score) was determined. Using this method, PD_AR_ subjects had a ratio ≤0.8, whereas PD_T_ subjects had a ratio ≥0.9. In the current study, the average ratio for PD_AR_ subjects was 0.18 ± 0.22 (range 0–0.73), vs. 2.63 ± 1.77 (range 0.9–7.14; Table 1) for PD_T_ subjects.

### 2.3. MRI Imaging

MRI data were acquired on a Siemens 3T MRI system (Magnetom Trio, Siemens Medical Solutions USA, Inc., Malvern, PA, USA) with an 8-channel phased array head coil. Imaging was carried out while PD subjects were on medication. The rs-fMRI data were acquired with the following parameters: TR/TE/FA = 2000 ms/30 ms/90°; FOV = 240 × 240 mm^2^; acquisition matrix = 80 × 80; number of slices = 34; slice thickness = 4 mm; and the number of repetitions = 240. A 3D MPRAGE image was also acquired for volumetric analysis and anatomical overlay. To ensure wakefulness during fMRI scanning, subjects were instructed to relax and keep their eyes open. Imaging protocol did not include collecting respiration or heart rate data. A high-resolution, T1-weighted, 3D gradient-echo sequence (MPRAGE sequence) was also acquired using the following parameters: TR = 2300 ms; TE = 2.98 ms; flip angle = 90°; FOV = 256 × 256 mm^2^; matrix size = 256 × 256; slice thickness = 1 mm (no slice gap); number of slices = 160; and voxel size = 1 × 1 × 1 mm^3^.

### 2.4. Olfactory Network

**Figure 1:**
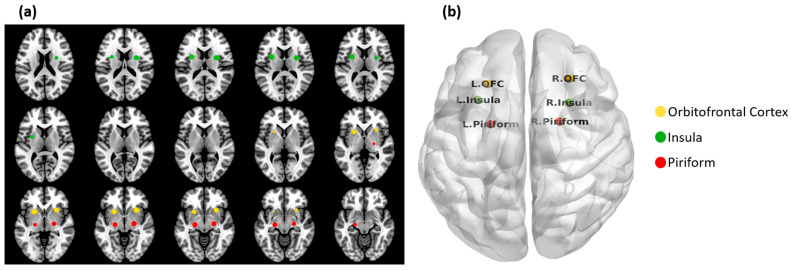
Brain regions in the ON in (**a**) axial slices and (**b**) in a 3D glass brain. These regions were selected based on published fMRI activation studies [31]. Time courses were extracted in Montreal Neurological Institute (MNI) space [(x y z) coordinates] from these six regions as an average time course within a five voxel radius centered on respective coordinates.

The coordinates of brain regions in the ON were selected based on a meta-analysis of fMRI task activation studies, identifying regions most likely to be activated by olfactory stimulation [31]. Figure 1 shows the ON, which includes the piriform cortex (PC) [(−22 0 −14) (22 2 −12)], insula [(−30 18 6), (28 16 8)], and orbitofrontal cortex (OFC) [(−24 30 −10) (28 34 −12)] [31]. These regions of interest (ROIs) in the ON have been shown to have the highest likelihood of being activated during olfactory stimulation [31]. Using AFNI’s 3dDeconvole, time courses from these seed regions were used to identify ON FC, including remote brain structures that comprised the extended ON. Functional connectivity proximal to seed regions was used to identify the core ON [32,33].

### 2.5. Functional Connectivity Analysis

Rs-fMRI data pre-processing and statistical analyses were performed using DPARSFA V4.5, implemented in DPABI (http://rfmri.org/DPARSFA (accessed on 2 June 2024)). In brief, the methods entailed the following: (1) the removal of the first 10 time points; (2) slice time correction; (3) realignment; (4) unified segmentation using 3D-T1 images and spatial normalization using the deformation parameters; and (5) time course de-trending and spatial smoothing.

The same software was used to estimate the motion parameters for all subjects, ensuring that maximum translation did not exceed 2.5 mm in any xyz direction or 3.0° rotation about any axis. One healthy control (HC) and one PD_T_ participant had maximum translations between 2.5 and 3.0 mm; however, since respiration and heart rate data were not collected, these subjects were excluded from the FC analysis. Seed-based FC analysis, as described in Tobia et al. (2016) [32], was employed to identify differences in ON connectivity between PD_AR_ and PD_T_ groups. Seed time courses were extracted from preprocessed rs-fMRI data normalized to the Montreal Neurological Institute (MNI) space, averaging signals within a five-voxel radius around the PC, insula, and OFC coordinates. These time courses identified correlated brain regions both proximal (core ON) and distal (extended ON) to the seeds. ON FC maps were corrected using AlphaSim in AFNI, with a voxel-level threshold *p* < 0.001 and cluster-level *p* < 0.05.

### 2.6. Voxel-Based Morphometry

Voxel-based morphometry (VBM) was performed using SPM12 r7771, accessed on 2 June 2024 (http://www.fil.ion.ucl.ac.uk/spm/). We performed the following steps: (1) inspected T1-weighted anatomical images to ensure no gross anatomical abnormalities; (2) segmented images into gray matter (GM), white matter (WM), and cerebrospinal fluid (CSF); (3) spatially normalized the segmented images—the image intensity of each voxel was modulated by Jacobian determinants to ensure that regional differences in the total amount of GM volume was conserved—(4) transformed registered images into the MNI space using the affine spatial normalization—during this normalization step, images were Jacobian-scaled for “modulated” VBM and resampled to 1.5 mm^3^ isotropic voxels—(5) the normalized and modulated GMV images were smoothed with an 8 mm full-width, at half-maximum (FWHM), isotropic Gaussian kernel; and (6) a one-way analysis of variance (ANOVA) was performed to determine volumetric group differences.

### 2.7. Statistical Analysis

Demographic (age and education) and clinical factors (HAM-D, MMSE, LEDD, and UPDRS III) were compared using a simple one-way ANOVA. The sex ratio between groups was compared using Fisher’s Exact Test. We used standardized neuropsychological testing to evaluate the overall cognitive health of PD subjects in five cognitive domains [34], namely in executive function, spatial memory, verbal memory, attention, and working memory: A detailed analysis of cognitive scores for this cohort is presented in Karunanayaka et al. (2016) [22]. Briefly, a one-way analysis of covariance (ANCOVA) was used to examine group differences while controlling for HAM-D scores. Similarly, group differences were examined using one-way ANCOVA and the Tukey–Kramer method was used to correct for multiple comparisons. See Table S2 in the Supplementary Materials for details.

### 2.8. Multivariate Classification of ON FC Values

We examined whether FC patterns within the ON contain discriminative information that can differentiate between PD_AR_ and PD_T_ subtypes. A multivariate classification approach was implemented by combining a principal components analysis (PCA) of ON FC values across regions with a nearest-neighbor classifier using the Classify function in Mathematica (Wolfram, Champaign, IL, USA). This embedding-based method integrates a neurobiologically informed model of ON function with a machine learning-based classification. Compared to traditional correlation-based FC approaches, such multivariate techniques can better capture subtle, distributed connectivity differences. As demonstrated by Brodersen et al. (2011), these methods exploit discriminative information encoded in latent physiological variables—such as synaptic connectivity strength—to improve network classification accuracy [35].

### 2.9. Data Availability

The data cannot be made available as no patient approval has been obtained for sharing coded data. However, the anonymized data and output files of the analyses will be made available on request.

## 3. Results

### 3.1. Demographic and Cognitive Comparisons

There were no significant differences in age, MMSE, sex ratio, or education between PD_AR_, PD_T_, and CN subjects (*p* > 0.068; see Table 1 and Table S1 in Supplementary Material). PD subjects had significantly higher UPDRS III scores (*p* < 0.0001) compared to CN. PD_AR_ subjects had significantly higher HAM-D scores than CN. The five cognitive domains showed no differences among the three groups after FDR correction (*p* > 0.075). Details are given in Table S2 of the Supplementary Material.

### 3.2. UPSIT Group Differences

**Figure 2:**
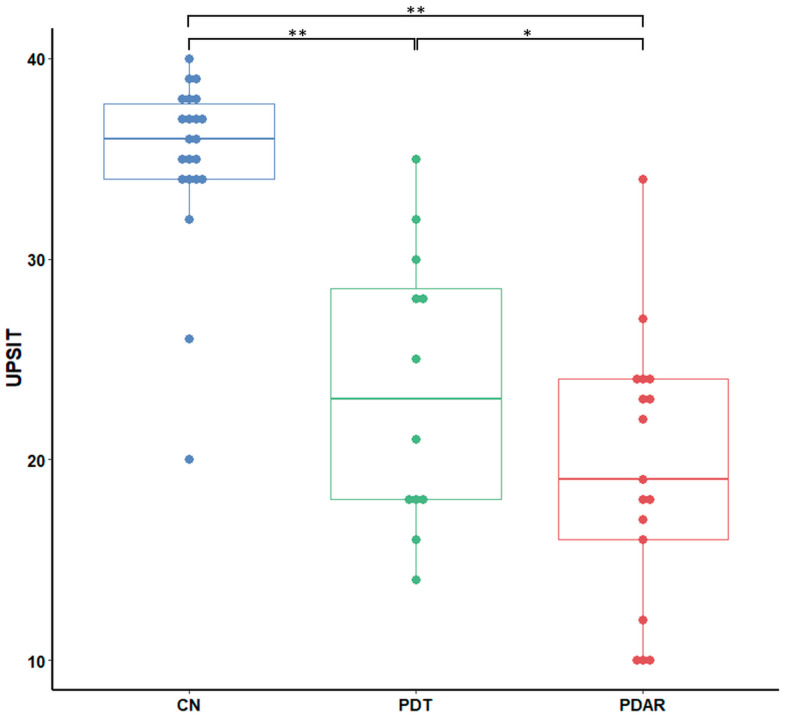
Group differences in UPSIT scores (mean ± SD) between CN, PD_T_, and PD_AR_. CN: 36.41 ± 2.15; PD_T_: 23.58 ± 6.96; PD_AR_: 19.47 ± 6.65. *, *p* < 0.05; ** *p* < 0.01.

The ANCOVA analysis detected group differences in UPSIT scores (F = 54.21, *p* < 0.001). Figure 2 provides the results of a post hoc analysis showing group differences in UPSIT scores. CN showed significantly higher UPSIT scores compared to PD subtypes. PD_AR_, however, had significantly lower UPSIT scores than PD_T_.

### 3.3. Volume Differences

VBM differences between groups were not detected after correcting for multiple comparisons with family-wise error (FWE) in SPM12. Differences were detected within the supplementary motor area with a statistical threshold of *p* < 0.001, uncorrected. A detailed analysis of GM volumes in respective groups is provided in Karunanayaka et al. (2016) [22]. A sample size calculation revealed that our study may be underpowered to detect significant GM group differences between CN and PD.

### 3.4. ON FC Group Differences

**Figure 3:**
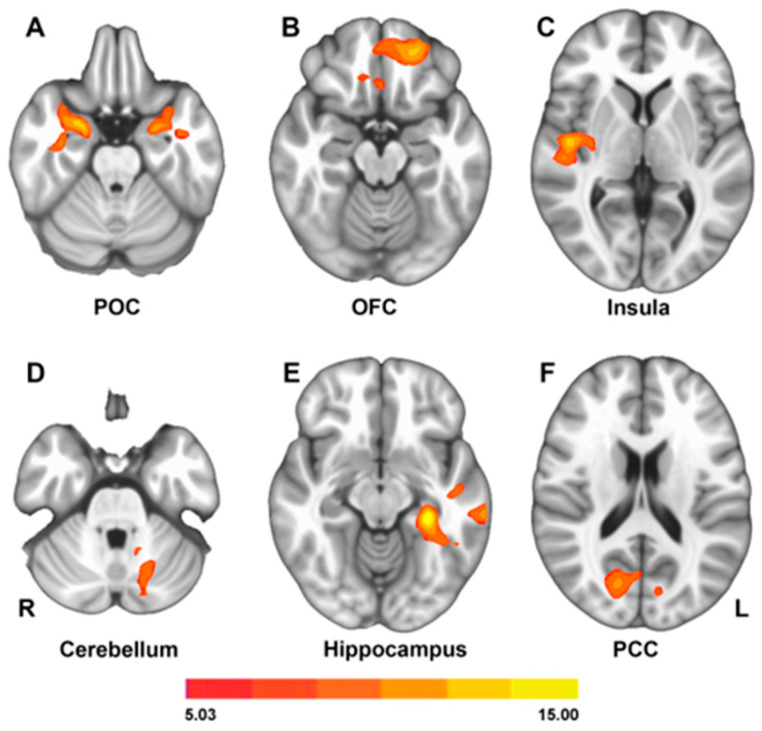
Group differences in ON FC between CN, PD_AR_, and PD_T_ groups. These brain regions have been implicated in olfactory processing in previous studies [31]. Significant clusters are displayed at *p* < 0.01, AlphaSim corrected. The heatmap represents t-values reflecting the magnitude of between-group differences in ON FC.

To investigate the neural basis of the UPSIT score differences shown in Figure 2, we conducted a whole-brain one-way ANOVA on ON FC values for each group. The primary olfactory cortex (POC), OFC, insula, cerebellum, hippocampus, and posterior cingulate cortex (PCC) showed ON FC group differences (Figure 3).

**Figure 4:**
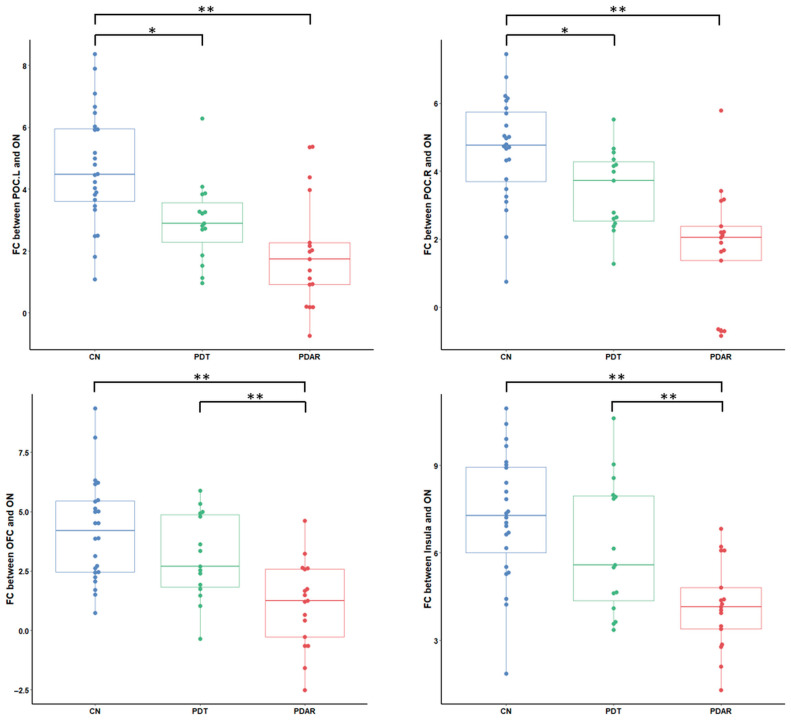
ON FC group differences within the core ON. The ON FC to the OFC and insula significantly differs between PD_AR_ and PD_T_. *, *p* < 0.05; **, *p* < 0.01.

**Figure 5:**
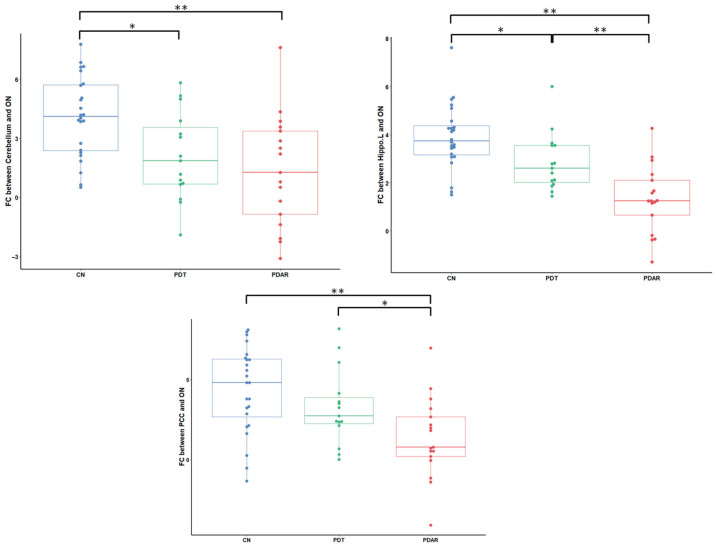
ON FC differences within the extended ON. The FC of the left hippocampus and the PCC significantly differs between PD_AR_ and PD_T_. *, *p* < 0.05, **: *p* < 0.01. Cere, Cerebellum; Hipp, Hippocampus.

We extracted ON FC values within the six brain regions identified in Figure 3 and investigated their group differences, which are highlighted in Figure 4 and Figure 5. In the core ON shown in Figure 4, POC connectivity to the ON differed significantly between the CN and both PD subtypes. In contrast, both OFC and insula connectivity to the ON was significantly different between PD_AR_ and CN as well as between PD_AR_ and PD_T_. These results support differential ON FC patterns between PD_T_ and PD_AR_ within the core ON.

Another pattern of differential ON FC was observed within the extended ON. As shown in Figure 5, ON FC between the left hippocampus and the PCC significantly differed between PD_T_ and PD_AR_. Note that the left hippocampus FC to the ON differed among all groups.

### 3.5. Correlations Between ON FC and UPSIT Scores

**Figure 6:**
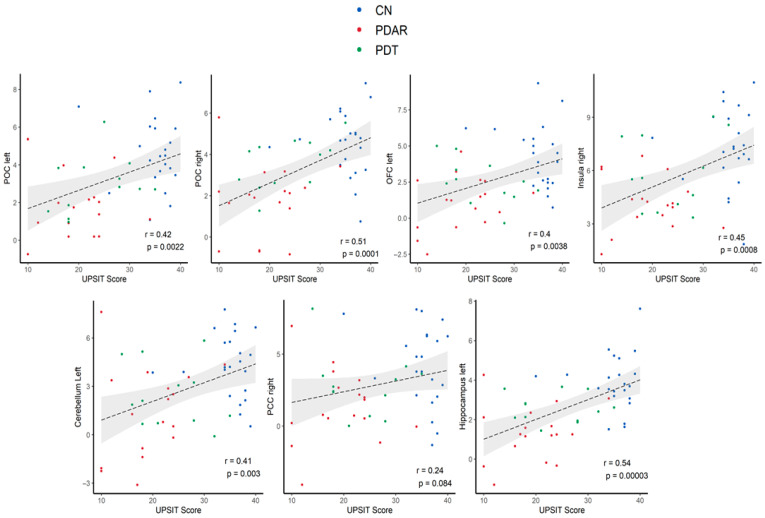
The correlations between UPSIT scores and the FC of each brain region within the ON were examined. Among the PD subtypes, PD_AR_ exhibited the lowest ON FC and UPSIT scores. Given that PD_AR_ is associated with a poorer prognosis, these findings suggest that ON FC and UPSIT performance may have potential as indicators of disease severity.

ON FC values within the six brain regions identified in Figure 3 did not correlate with UPSIT scores within individual groups. However, as shown in Figure 6, ON FC across core and extended ON regions was positively correlated with UPSIT scores in the combined sample. When group effects were considered, the correlations became insignificant. Thus, as shown in Figure 6, the correlations are likely driven by group differences between CN and PD participants. Additionally, Figure 6 shows subtype-dependent clustering: PD_AR_ participants showed the weakest ON FC and lowest UPSIT scores.

### 3.6. Multivariate Classification of ON FC

**Figure 7:**
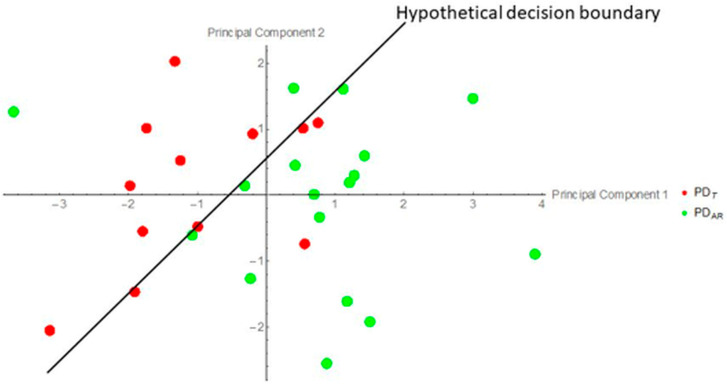
Multivariate ON FC data projected onto the first two principal components. In this reduced plane, a decision boundary can be identified, separating PD_AR_ (17) and PD_T_ (12) at above-chance levels.

Multivariate classification analysis was performed in Mathematica to determine whether ON FC values contained sufficient information, above chance level, to distinguish between PD_AR_ and PD_T_ subtypes. Feature vectors consisted of the ON FC values from each group. Classification was implemented using the versatile Classify function in Mathematica (see Classify—Wolfram Language Documentation for details). The analysis demonstrated that ON FC values provided above-chance discriminative information (classification accuracy > 50%) for separating PD_AR_ from PD_T_, suggesting that ON FC can serve as a potential neural marker for differentiating Parkinson’s disease subtypes. Before performing the nearest-neighbor classification analysis, we performed PCA on the multivariate ON FC data, as shown in Figure 7. The classification accuracies were 67% for PD_T_ and 83% for PD_AR_. As such, these results provide convincing evidence to suggest that ON FC in fact contains above-chance discriminatory information for distinguishing between the two PD subtypes.

## 4. Discussion

Olfactory dysfunction is highly prevalent in neurodegenerative diseases and often emerges in the preclinical stage, persists throughout disease progression, and is easily testable—making it a promising marker for early diagnosis, differential diagnosis, and prognosis. PD-related olfactory dysfunction is believed to be due to alpha-synuclein pathology in the OB and anterior olfactory nucleus, consistent with Braak staging of PD [12,36,37,38]. This suggests that PD pathology may begin in the primary olfactory structures and the lower brainstem before reaching higher-order olfactory structures and the substantia nigra.

This study provides strong evidence for dissociable impairments in ON FC between PD_T_ and PD_AR_ subtypes of PD. Aside from the olfactory performance, the subtypes were well-matched for age, demographic variables, and cognitive performance. Prior clinical studies have documented differences in cortical and subcortical networks affected in these subtypes, which may contribute to variations in disease progression and cognitive decline [8,26].

In line with these findings, PD_AR_ participants—known to experience faster motor and cognitive deterioration—exhibited lower UPSIT scores and reduced ON FC strength compared to PD_T_, despite being matched on clinical measures such as UPDRS-III, the Hoehn and Yahr scale, levodopa dose, disease duration, and cognitive status [9]. ON FC likely reflects the temporal coordination of activity across anatomically distributed olfactory regions, and such functional interactions appear to underlie olfactory performance [39]. These subtype-specific ON FC patterns highlight central olfactory system deficits in PD and reinforce the presence of distinct neuropathological mechanisms in PD_AR_ versus PD_T_ [8,40].

### 4.1. Impact of Non-Olfactory Cognitive Scores on UPSIT

Olfaction is a multidimensional sense that can be influenced by other cognitive domains, such as memory, verbal processing, and attention [41]. However, in the present study, the three groups were closely matched in age, demographic characteristics, and cognitive performance. This careful matching minimizes the potential confounding effects of non-olfactory cognitive factors on UPSIT scores. Furthermore, the ON FC group differences shown in Figure 3 involve regions implicated in higher-order olfactory processing—for example, the hippocampus—highlighting the well-established relationship between olfaction and memory function [24,42]. These findings suggest that the observed group differences are likely attributable to genuine differences in olfactory function. Specifically, higher ON FC appears to be associated with a better olfactory performance.

### 4.2. ON FC Distribution

We observed a positive correlation between ON FC and UPSIT scores across the combined sample, suggesting a meaningful link between ON FC and olfactory performance [43]. While within-group correlations were not specifically quantified, the combined analysis (Figure 6) revealed subtype-dependent clustering: PD_AR_ participants showed the weakest ON FC and lowest UPSIT scores. However, once group effects were accounted for, the bivariate correlations lost statistical significance, suggesting that the observed association was primarily driven by PD subtype differences. This may also reflect limitations due to the relatively small sample sizes. Despite this, the observed trends support the potential for ON FC to reflect pathological changes in key olfactory regions such as the POC and hippocampus, as well as behavioral deficits in PD. Similar associations have been proposed in Alzheimer’s disease using ON FC as a marker of olfactory dysfunction [33]. Our findings offer in vivo evidence for central ON involvement—beyond the OB and tract—in PD, consistent with the view that olfactory impairments in PD arise from disrupted central processing [44].

Olfactory deficits are common in early PD and currently serve as supportive diagnostic indicators. Often emerging as a prodromal symptom, smell loss frequently precedes motor and cognitive decline. For instance, the reduced intrinsic integrity of the substantia nigra in individuals with idiopathic olfactory loss has been used to confirm a PD “at-risk” status [45]. Conversely, patients who are normosmic and cognitively intact at diagnosis tend to show a stable cognitive performance for up to a decade [46]. Additionally, PD-related olfactory impairments have been shown to help differentiate PD from other movement disorders [47,48].

Our results suggest that the preferential involvement of the ON in PD subtypes—PD_T_ and PD_AR_—may lead to measurable differences in ON FC even before cognitive impairments emerge [22]. This raises the possibility that ON FC could serve as a prognostic marker for identifying individuals at risk for cognitive decline, such as progression to MCI in PD. Moreover, ON FC may reflect the integrity of functional brain communication within the olfactory system, offering a potential indicator of disease severity [39]. These findings reveal a novel brain–behavior relationship, underscoring the close correspondence between motor and olfactory impairments in PD.

Motor symptoms in PD result from the loss of dopaminergic neurons in the nigrostriatal pathway [49]. In contrast, PD-related olfactory deficits do not improve with dopaminergic treatment and are believed to arise from extranigral pathology [50]. Our findings indicate that olfactory and motor impairments may share a common neural substrate at the network level, as evidenced by the rs-fMRI data. We hypothesize that dysfunction in this shared substrate may increase the risk of developing PD dementia, given the strong link between olfaction and cognition.

Unlike Zhou et al. (2019), who estimated FC of individual olfactory brain regions [51], our study focused on the overall connectivity of the ON, combining both common and individual FC patterns. Our network-level approach aimed to demonstrate that group differences in UPSIT scores correspond to connectivity changes within the ON. Nonetheless, we acknowledge that Zhou et al.’s method could serve as an alternative way to investigate the neural basis of UPSIT group differences in our data [51].

### 4.3. Network Perspective of Olfactory Function in PD

Localized brain functions are thought to arise from distributed global connectivity patterns [52]. Extending this principle to olfaction, our study suggests that ON FC may provide a mechanistic framework for understanding PD-related olfactory impairments. Identifying how PD pathology disrupts brain function is critical for developing early diagnostic tools and interventions to slow disease progression. Notably, olfactory regions are among the first affected by pathological protein aggregates [53,54], making the ON particularly vulnerable to early neurodegeneration. This supports our decision to include cortical brain regions in the ON that are most likely to be activated by olfactory stimulation. However, as mentioned earlier, the pathology in the OB—which has strong bidirectional connections with the POC—is common in early PD [55]. Although the OB is difficult to image with 3T fMRI and was not included in this study, the consistent presence of olfactory deficits across neurodegenerative diseases may reflect the disruption of a shared, evolutionarily conserved neural substrate.

### 4.4. Resting-State fMRI

RS brain networks are intrinsically organized and modulated by the task performance [52,56], and a strong correspondence between RS functional connectivity (RSFC) and task-evoked connectivity has been demonstrated, including in the olfactory system. RSFC also closely aligns with structural brain connectivity [57]. In this study, we examined whether ON FC differs between the core and extended ON in PD subtypes. While no significant differences in ON FC to the POC were observed, PD_AR_ participants exhibited significantly reduced ON FC to the OFC and insula—key multisensory integration hubs. Additionally, connectivity to the hippocampus and PCC within the extended ON was significantly lower in PD_AR_. Notably, ON FC to the hippocampus mirrored patterns in the UPSIT scores (Figure 3), aligning with findings by Westermann et al. (2008) suggesting hippocampal involvement in olfactory sensitivity [58]. These results reveal widespread ON FC alterations in PD and support the hypothesis that resting-state connectivity disruptions underlie olfactory deficits in PD [59].

### 4.5. Gray Matter Comparison

In PD, the relationship between ON atrophy and olfactory impairment remains unclear [60]. Some studies have reported cortical GM loss—such as atrophy in the right piriform cortex—to be associated with olfactory deficits in PD. However, other investigations have failed to identify consistent relationships between GM atrophy and olfactory impairment, highlighting the need for further research to clarify the structural basis of olfactory dysfunction in PD [61,62,63]. The present study examined cortical atrophy within the ON and its relationship with olfactory function in a relatively large sample of early-stage PD patients. We did not observe statistically significant GM volume loss within the ON, suggesting that regional atrophy may be less pronounced in the absence of comorbid cognitive impairment. However, this absence should not be interpreted as evidence that PD has no impact on the GM structure. Rather, our findings suggest that functional connectivity changes in the ON may be more sensitive than structural changes, particularly in early stages of the disease. Given that olfactory deficits are predictive of dementia in PD, ON FC may serve as a promising biomarker for identifying individuals at elevated risk of cognitive decline [64].

### 4.6. Multivariate ON FC

Our findings demonstrated that multivariate ON FC measurements—specifically the first two principal components—contain sufficient discriminative information to distinguish between PD_AR_ and PD_T_ subtypes. The PCA revealed distinct ON FC patterns across regions, enabling the accurate classification of the two groups and pointing to underlying differences in neural mechanisms. While the classifier accuracy offers insight into discriminative power, it should not be interpreted as a direct measure of the effect size. Accuracy can increase when data are aggregated within groups, but this often comes at the cost of reduced statistical power due to smaller sample sizes. Future work should explore integrated approaches that combine multivariate and univariate analyses to better understand the brain mechanisms underlying PD subtypes.

### 4.7. Study Limitations and Future Directions

Given the small sample size of our study, larger cross-sectional and longitudinal investigations are needed to validate the integrity of ON FC and clarify its relationship with motor and olfactory impairments in PD. Complex neurodegenerative changes may occur across brain networks even in PD patients with mild cognitive impairment (PD-MCI), and characterizing olfactory dysfunction in this group is crucial for understanding PD pathophysiology. Such insights could inform early intervention strategies to delay or prevent the onset of PD dementia. Additionally, the framework presented here could be extended by integrating resting-state FC with task-based FC measures—such as those derived from odor threshold, discrimination, or identification paradigms—to develop a unified FC metric for better characterization of PD subtypes.

Although we initially defined a simplified ON consisting of six core regions, our functional connectivity analysis revealed the recruitment of additional brain areas, including the hippocampus, cerebellum, and PCC—regions that are well-documented to play key roles in both olfactory and memory-related processing [24,31,42,65]. Therefore, in line with Zhou et al. (2019), future research should focus on connectivity within an expanded ON, which represents a valuable extension of our current approach [32,33]. We expect that incorporating enriched ON FC features will be particularly useful for developing more sensitive classifiers to distinguish PD_AR_ from PD_T_.

Since PD is characterized by heterogeneous symptoms and progression patterns, subtype identification has become a critical focus of current research. For instance, the side of motor symptom onset (left or right) has been shown to influence disease progression and the manifestation of specific non-motor symptoms. Subtype classification can also be based on prominent non-motor features such as depression, anxiety, rapid eye movement (REM) sleep behavior disorder (RBD), and olfactory dysfunction. Furthermore, growing evidence suggests that the classification of motor subtypes in PD is not static and may shift over time with disease progression duration. Therefore, it is essential to integrate and build upon current findings from studies focused on various PD subtypes to improve disease characterization and targeted interventions.

Although olfactory impairment is one of the earliest symptoms of PD, often preceding the onset of motor signs and cognitive decline, it is also prevalent in other neurodegenerative disorders and, therefore, lacks specificity [47]. As such, it is best utilized in combination with other prodromal markers of PD—such as REM sleep behavior disorder and constipation [66,67]—to enhance early detection strategies.

## 5. Conclusions

Alterations in olfaction were associated with distinct ON FC patterns in PD subtypes. Compared to CN and PD_T_ patients, the PD_AR_ subtype showed greater ON FC impairments, despite groups being well-matched for age, demographic, and clinical variables, as shown in Table 1. These differences persisted even after adjusting for the gray matter volume and cognitive performance (MMSE). The data revealed a strong association between olfactory and motor symptoms in PD and suggest that olfactory dysfunction may serve as a novel biomarker for early PD severity. Previously, using this cohort, we demonstrated that posterior DMN impairments in PD_AR_ may contribute to an increased risk of cognitive decline [22]. Our current findings similarly show greater ON FC deficits in PD_AR_, supporting the hypothesis that olfactory impairment may predict cognitive vulnerability in PD [68]. Future studies with larger cohorts and longitudinal designs are needed to validate these findings and explore whether combining ON and DMN metrics enhances the prognostic accuracy [69,70]. Overall, this study highlights olfactory impairment as a promising clinical biomarker of PD severity and progression.

## Figures and Tables

**Table 1:** Demographic information of the study cohort (mean ± standard deviation). *p*-values were derived from Fisher’s Exact test for gender. All other comparisons, age, education, Mini-Mental State Examination (MMSE), and Hamilton Depression Rating Scale (HAM-D Scale), were performed using one-way ANOVA. Statistical significance was evaluated using *p* < 0.05. Other abbreviations: UPDRS = Unified Parkinson’s Disease Rating Scale; T/AR = mean tremor/mean akinetic rigidity score; LEDD = Levodopa Equivalent Daily Dose; H&Y stage = Hoehn and Yahr; NA = Not Available, * = *p*-value < 0.05.

	CN (n = 24)	PD_AR_ (n = 17)	PD_T_ (n = 15)	*p*-Value		
				CN vs. PD_AR_	CN vs. PD_T_	PD_AR_ vs. PD_T_
Age (yrs.)	57.8 ± 7.4	59.1 ± 7.4	61.7 ± 6.7	0.836	0.236	0.569
Gender (M:F)	11:13	9:8	6:9	0.876	0.423	0.724
Education (yrs.)	15.8 ± 2.4	13.9 ± 2.1	15.2 ± 3.2	0.068	0.795	0.329
MMSE	29.6 ± 0.9	29.1 ±1.2	29.7 ± 0.5	0.257	0.959	0.221
HAM-D Scale	4.2± 2.7	7.2 ± 3.3	6.9 ± 4.0	0.0003 *	0.078 *	0.215
Disease duration (yrs.)	NA.	3.7 ± 4.6	3.5 ± 3.5	NA.	NA.	0.91
UPDRS III	1.2 ± 2.6	20.8 ± 12.9	17.1 ± 9.5	<0.0001 *	<0.0001 *	0.48
T/AR	NA.	0.18 ± 0.22	2.63 ± 1.77	NA.	NA.	<0.0001 *
H&Y stage	NA.	1.7 ± 0.6	1.7 ± 0.6	NA.	NA.	0.06
LEDD (mg/day)	NA.	366 ± 229	354 ± 282	NA.	NA.	0.88

## Data Availability

The data generated and analyzed during the current study are not publicly available since they were purposively collected by the authors for the present study, but they are available from the corresponding author upon reasonable request.

## Supplementary Materials

The following supporting information can be downloaded at: https://www.mdpi.com/article/10.3390/life15081324/s1, Table S1: Full summary of UPDRS scores for PD_AR_ and PD_T_. AR indicates akinetic-rigidity subtype. T indicates tremor-predominant subtype, Table S2: Neuropsychological test scores and analyses.
