# Supplementary material for: Olfactory Network Functional Connectivity as a Marker for Parkinson’s Disease Severity
Source: Life (Basel). 2025 Aug 20;15(8):1324. doi: 10.3390/life15081324 (PMC12387693; doi:10.3390/life15081324)
Supplement: Supplementary file 1 [file life-15-01324-s001.zip › life-3777118-supplementary.pdf]

PD subtypes	Total tremor scores	Mean Tremor score	Total AR scores	Mean AR score	Mean Tremor/AR ratio
**PD$_{AR}$ patients**					
4	0	0.00	10	0.71	0.00
7	4	0.44	11	0.79	0.57
16	1	0.11	8	0.57	0.19
22	0	0.00	15	1.07	0.00
30	1	0.11	11	0.79	0.14
36	0	0.00	30	2.14	0.00
45	10	1.11	33	2.36	0.47
59	6	0.67	11	0.79	0.85
67	4	0.44	18	1.29	0.35
68	0	0.00	16	1.14	0.00
73	0	0.00	6	0.43	0.00
92	5	0.56	17	1.21	0.46
104	5	0.56	26	1.86	0.30
129	3	0.33	23	1.64	0.20
183	0	0.00	10	0.71	0.00
187	0	0.00	6	0.43	0.00
190	0	0.00	30	2.14	0.00
**PD$_T$ patients**					
23	6	0.67	6	0.43	1.56
31	7	0.78	9	0.64	1.21
40	3	0.33	3	0.21	1.56
54	6	0.67	5	0.36	1.87
74	11	1.22	12	0.86	1.43
81	3	0.33	4	0.29	1.17
85	3	0.33	5	0.36	0.93
87	5	0.56	1	0.07	7.78
94	7	0.78	3	0.21	3.63

100	11	1.22	7	0.50	2.44
105	4	0.44	5	0.36	1.24
111	4	0.44	6	0.43	1.04
178	7	0.78	6	0.43	1.81
181	9	1.00	15	1.07	0.93
182	5	0.56	4	0.29	1.94

Table S1. Full summary of UPDRS scores for PD_{AR} and PD_{T}. AR indicates akinetic-rigidity subtype. T indicates tremor-predominant subtype.

Standardized neuropsychological testing for PD subjects in five cognitive domains

Cognitive test scores were converted to standardized z-scores. One-way analysis of covariance (ANCOVA) was used to examine group differences in each cognitive domain with adjustments for HAM-D scores—where the individual cognitive domain was the dependent variable and the group was the independent variable. One-way ANCOVA was also used to test for group differences in individual cognitive tests with HAM-D scores as a covariate. The Tukey-Kramer method was used to correct for multiple group (3 groups) comparisons. Since multiple cognitive tests were compared, results were corrected for multiple comparisons of five test domains with a false discovery rate (FDR) at the 0.05 level. We report raw p values but indicate whether the results were also significant after FDR correction at a p value of 0.05.

Test	CN (*n = 24*)	PD_{AR} (*n = 17*)	PD_T (*n = 15*)	*P*-value HC vs. PD_{AR}	HC vs. PD_T	PD_{AR} vs. PD_T
Executive function						
Z-scores	0.39 ± 0.40	0.05 ± 0.59	0.09 ± 0.37	0.075	0.223	0.788
CWInt Inhibition	0.65 ± 0.70	0.29 ± 1.04	0.53 ± 0.85	0.866	0.999	0.882
Inhibition Errors	0.01 ± 0.76	-0.49 ± 1.03	0.33 ± 0.58	0.409	0.360	0.032
CWInt Switch	0.65 ± 1.00	-0.08 ± 1.34	0.62 ± 0.63	0.226	0.998	0.202
Switch Errors	0.46 ± 0.64	-0.08 ± 0.91	0.47 ± 0.57	0.195	0.976	0.133

VVT_Total	0.20 ± 0.80	-0.21 ± 1.22	-0.33 ± 1.68	0.971	0.661	0.826
VVT_Switch	0.05 ± 0.87	-0.36 ± 1.15	-0.59 ± 1.77	0.978	0.524	0.686
DesFlu Switch	0.69 ± 0.89	0.55 ± 0.70	0.22 ± 0.91	0.786	0.204	0.600
DesFlu Total Correct	0.82 ± 1.18	0.12 ± 0.76	0.18 ± 0.81	0.286	0.234	1.00
DesFlu Total Design	1.07 ± 1.30	0.59 ± 0.85	0.80 ± 1.42	0.739	0.897	0.943
DesFlu Design Accuracy	-0.36 ± 0.92	-0.75 ± 1.12	-0.80 ± 1.43	0.531	0.452	0.998
VerbFlu Letter	0.01 ± 0.85	-0.43 ± 1.14	-0.41 ± 0.85	0.101	0.191	0.900
VerbFlu Category	0.46 ± 0.92	0.29 ± 0.90	0.08 ± 1.02	0.429	0.245	0.959

<u>Spatial cognition</u>

Z scores	0.15 ± 0.71	-0.31 ± 0.79	-0.03 ± 0.58	0.183	0.755	0.505
JoLO	0.30 ± 1.42	-0.51 ± 1.50	0.05 ± 1.17	0.252	0.742	0.632
DRS2 Construction	0	-0.12 ± 0.33	0	0.243	0.997	0.214
<u>Learning/Memory</u>						
**<u>Spatial Memory</u>**	0.03 ± 0.84	-0.61 ± 1.35	-0.41 ± 1.48	0.695	0.768	0.985
BVMT Learning	0.00 ± 1.10	-0.51 ± 1.24	-0.48 ± 1.40	0.828	0.693	0.981
BVMT Delayed Recall	0.21 ± 1.24	-0.27 ± 1.20	-0.01 ± 1.32	0.975	0.999	0.977
BVMT Discrimination Index*	-0.10 ± 1.29	-1.05 ± 2.24	-0.74 ± 2.14	0.558	0.715	0.951

**Verbal Memory**	-0.57 ± 1.15	-0.55 ± 1.00	-0.43 ± 0.66	0.982	0.959	0.898
HVLT Total Learning	0.49 ± 1.15	-0.64 ± 1.03	-0.78 ± 0.92	0.717	0.571	0.983
HVLT Delayed Recall	-0.65 ± 1.25	-0.68 ± 1.09	-0.42 ± 0.98	0.967	0.877	0.760
HVLT Discrimination Index*	-0.57 ± 1.47	-0.33 ± 1.31	-0.09 ± 0.62	0.896	0.544	0.846
Attention/working Memory						
Z-scores	0.62 ± 0.68	0.29 ± 0.50	0.41 ± 0.64	0.301	0.576	0.845
Letter Number Sequencing	0.43 ± 0.75	0.33 ± 0.88	0.09 ± 0.82	0.818	0.357	0.765
Spatial Span	0.44 ± 0.90	0.22 ± 0.90	0.44 ± 1.16	0.913	0.990	0.855
Digit Span	0.42 ± 0.68	0.20 ± 0.71	0.29 ± 0.74	0.301	0.642	0.791

Table S2*: Neuropsychological test scores and analyses.* *Neuropsychological test results showing the mean ± standard deviation. All test scores were converted to standard z-scores. Higher z-scores indicate better performance. One-way analysis of covariance (ANCOVA) was conducted with Group as the independent variable and each Cognitive Domain as the dependent variable, with the Hamilton Depression score serving as a covariate. Individual neuropsychological subtests were also tested using ANCOVA, with the Hamilton Depression score used as a covariate. Here, we report raw p-values. None of the test results were significant after FDR (false discovery rate) correction at a p value of 0.05. [CWInt = Color-Word Interference Test; VVT = Visual Verbal Test; DesFlu = Design Fluency; VerbFlu = Verbal Fluency; JoLO = Judgment of Line Orientation; BVMT-R = Brief Visuospatial Memory Test-Revised; HVLT-R = Hopkins Verbal Learning Test-Revised.]*

*Discrimination index scores are an index of recognition memory load, i.e., on number of Hits – number of False Alarms.
